# Commentary: Synaptic Excitation in Spinal Motoneurons Alternates with Synaptic Inhibition and Is Balanced by Outward Rectification during Rhythmic Motor Network Activity

**DOI:** 10.3389/fncir.2018.00001

**Published:** 2018-01-18

**Authors:** Rune W. Berg

**Affiliations:** Department of Neuroscience, University of Copenhagen, Copenhagen, Denmark

**Keywords:** spinal cord, central pattern generators (CPG), balanced networks, rhythm generation, motor control, patch-clamp, voltage-clamp

In a recent study, Guzulaitis and Hounsgaard (2017) (GH2017) used whole cell voltage clamp (VC) on the reversal potential for inhibition or excitation to assess their synaptic currents (Johnston and Wu, 1995; Brette and Destexhe, 2012). GH2017 concluded that inhibition and excitation alternated during rhythmic scratching, and a voltage-dependent intrinsic conductance was masking this input such that it appeared as balanced excitation and inhibition in previous published work (Berg et al., 2007; Petersen et al., 2014). Nevertheless, this reasoning relies entirely on the validity of the clamp and, as we will see below, there is a clamp error, which complicates the interpretation of their data. Errors associated with voltage-clamp is a common problem as noted in previous reports (Spruston et al., 1993; Williams and Mitchell, 2008; Petersen, 2017).

The membrane current (I) is composed of intrinsic, leak, excitatory and inhibitory currents with individual conductances and reversal potentials, which collectively form a membrane resistance (*R*_*m*_) and an equilibrium potential (*E*_*m*_). When recording these using a pipette electrode, its resistance (*R*_*s*_), sometimes called access or series resistance, is in series with *R*_*m*_ (Figure 1A). When there is no electrode current the membrane potential *V*_*m*_ = *E*_*m*_. However, during VC, a non-zero current introduces a drop in potential over *R*_*s*_, which can only be partially compensated with the amplifier electronics (Brette and Destexhe, 2012). *R*_*s*_ therefore has an uncompensated part (blue, *R*_*us*_, Figures 1A,B), which generates an unaccounted drop in potential from the clamp potential (*V*_*c*_) proportional to the pipette current:

(1) $$V_{m}=V_{c}-I\cdot R_{us}$$

GH2017 report: “Voltage clamp (VC) experiments were performed on motoneurons when access resistance was low (*R*_*a*_ < 20 MΩ) and possible to compensate by 60-80%.” This means that *R*_*us*_ = 20 − 40% · 20 MΩ = 4–8 MΩ. When clamping at 0 mV the applied current is likely large. The authors do not report *I* for their clamp experiments (Figures 8–9), but their IV-plots suggest up to 10 nA (Figures 5E, 6). Hence, when trying to clamp at 0 mV, *V*_*m*_ is really −10*nA*·4*MΩ* = −40*mV* with 80% *R*_*s*_-compensation.

**Figure 1:**
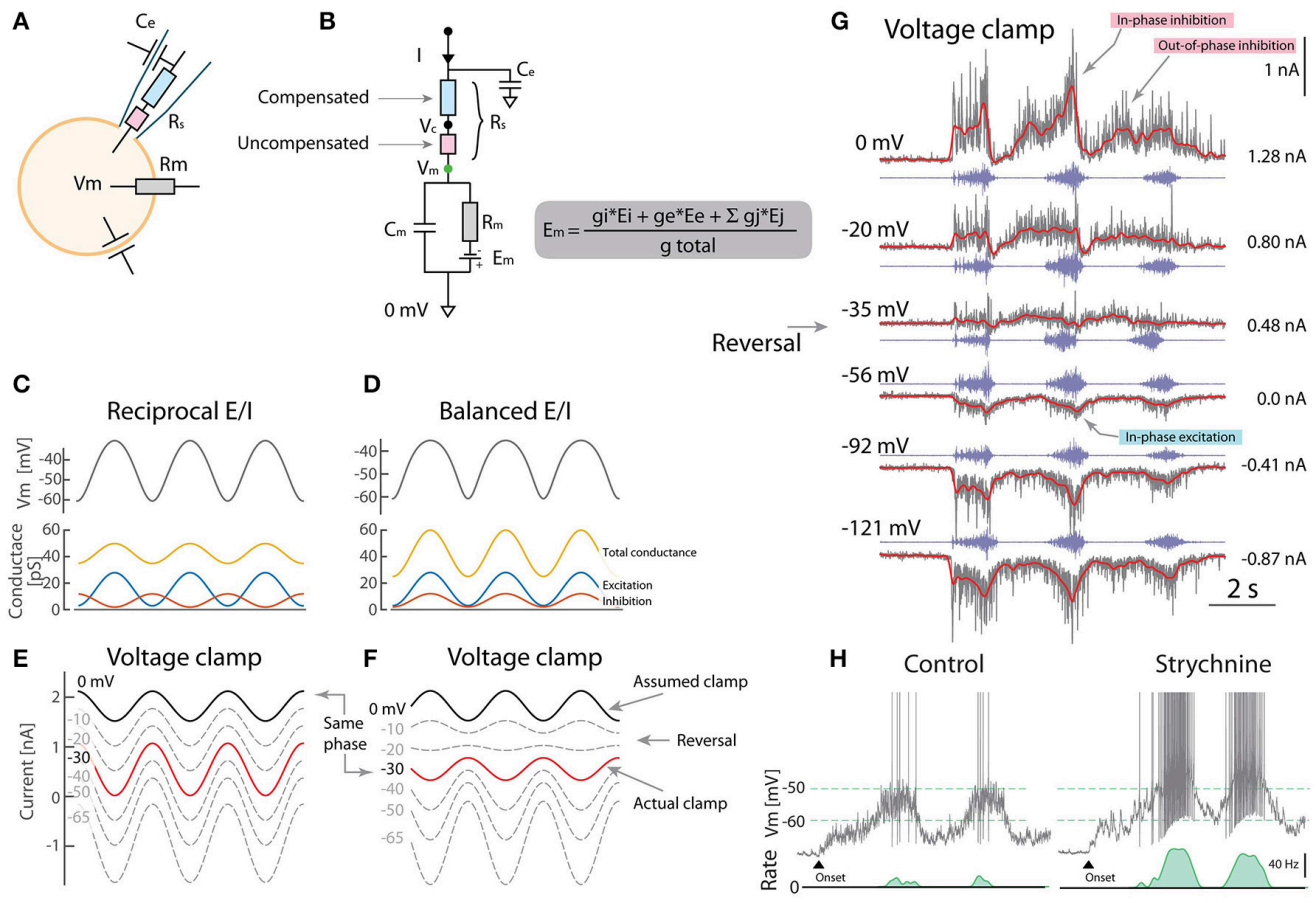
Caveats using voltage clamp to resolve excitation and inhibition. **(A)** Whole-cell VC can be decomposed into electrical components including the pipette series resistance (*R*_*s*_). **(B)** Partial compensation for *R*_*s*_ introduces a disparity between clamped potential (*V*_*c*_) and *V*_*m*_ due to uncompensated resistance (red). **(C)** Reciprocal model for rhythmic *V*_*m*_ has alternating E/I. (*R*_*m*_ = 20*MΩ*, *E*_*leak*_ = −70*mV*). **(D)** Balanced model has concurrent E/I and also rhythmic *V*_*m*_. **(E)** Outward currents measured using VC is assumed to be inhibition when clamping 0mV (black). The actual clamp is at −30 mV (red). **(F)** Balanced E/I spuriously appears as reciprocal when the actual clamp is below synaptic reversal potential (“same phase” cf. red in **F** and black in **E**). **(G)** VC-recording of a putative motoneuron with blocked spikes (with intracellular QX314) at different holding potentials (gray: current, red: mean, blue: nerve). Reversal of phase (arrow) is consistent with the balanced scheme **(F)** although with a smaller out-of-phase inhibition (indicated). **(H)** Blocking inhibition (strychnine) increases firing rate also consistent with the balanced scheme. **(G)** provided by *A. Alaburda* (current levels indicated, right) and **(H)** adapted with permission (Vestergaard and Berg, 2015).

To better understand the issue, we consider steady-state where all current passes through the resistors. From Ohm's law the voltage drop over *R*_*us*_ is *V*_*m*_ − *V*_*c*_ = *I* · *R*_*us*_. Similarly, the voltage drop over the membrane is *E*_*m*_ − *V*_*m*_ = *I* · *R*_*m*_. Combining these we can eliminate *I* and isolate *V*_*m*_:

(2) $$V_{m}=\frac{V_{c}R_{m}+E_{m}R_{us}}{R_{m}+R_{us}}$$

Hence, for a good clamp (*V*_*m*_ ≈ *V*_*c*_) it is required that *R*_*m*_ ≫ *R*_*us*_. GH2017 report a membrane conductance of 49.2 nS (Figure 5B), which gives *R*_*m*_ = 20*MΩ*. With these values (*E*_*m*_ = −70*mV*) clamping at 0 mV gives

(3) $$V_{m}=\frac{0-70mV\cdot 8M\Omega }{28M\Omega }=-20mV$$

Whereas *R*_*us*_ is assumed constant, *R*_*m*_ may change dramatically due to synaptic and intrinsic conductance. GH2017 nicely document a nonlinearity starting at −30 mV (Figures 5, 6), and a conductance of 314 nS (*R*_*m*_ = 3.2*MΩ*). Here, the low *R*_*m*_ even becomes smaller than *R*_*us*_ and therefore the clamp deteriorates further:

(4) $$V_{m}=\frac{0-70mV\cdot 8M\Omega }{11.2M\Omega }=-50mV$$

The clamp is unlikely to be this bad, since the reduction in *R*_*m*_ occurs above −50mV. Also, *E*_*m*_, which we assume constant, may depolarize due to change in the weighted average (Figure 1B), which mitigates the effect. The exact level of clamping of *V*_*m*_ with (*V*_*c*_ = 0mV) is difficult to estimate and may change in time. A reasonable guess is around *V*_*m*_ = −30*mV*.

What is the consequence of this clamping error? To address this question, we use a one-compartment model receiving either reciprocal (Figure 1C) or concurrent (balanced) excitation and inhibition (E/I) (Figure 1D), which are the schemes that GH2017 intended to distinguish between. Both result in rhythmic *V*_*m*_, although the effect of balanced E/I may seem counter-intuitive (Kolind et al., 2012; Petersen et al., 2014). The problem appears when presuming the outward current is inhibition, when setting *V*_*c*_ = 0mV (assumed clamp, black line Figures 1E,F). From the above, we know that the actual clamp is likely at −30 mV (red traces). Here, the phase of the outward current reverses making the actual clamp in the balanced scheme (red, F) appear qualitatively similar to the assumed clamp in the reciprocal (black, E). Therefore, the VC experiments by GH2017 are difficult to interpret and ill-suited to discriminate between these schemes.

Although reciprocal E/I is a widely held belief in the literature, there is remarkably little experimental support in tetrapod vertebrates. The Ia-inhibitory interneuron has reciprocal activity (Geertsen et al., 2011), whereas the Renshaw interneuron has recurrent inhibition, both connected to motoneurons. Nevertheless the action of the remaining inhibitory population is largely unexplored. The scarcity in experimental reports that resolve E/I input is likely due to nonlinear properties and difficulties in separating synaptic current, although methods have been proposed (Berg and Ditlevsen, 2013; Vich et al., 2017). Space clamp issues also confounds the separation of E/I (Chadderton et al., 2014). Previous observations in turtles based on current-clamp indicated concurrent E/I. Here, voltage-activated conductances were circumvented by injecting negative current to hyperpolarize *V*_*m*_ below the onset of the IV-nonlinearity. Therefore the disparity between reports cannot be attributed to outward rectification, as otherwise suggested by GH2017, see e.g., Figure 3A in Berg et al. (2007) and Figures 2–4 in Berg et al. (2008). Further, VC experiments were performed using sharp electrodes where spikes were blocked by pharmacology (QX314). QX314 likely also has the advantage of increasing *R*_*m*_, thus improving the *R*_*m*_ ≫ *R*_*us*_ requirement (Monier et al., 2008). A current-reversal was observed in accordance with the balanced scheme (Figure 1G). Other experiments confirm that when blocking excitation and inhibition pharmacologically, the high conductance vanish even at the same *V*_*m*_, suggesting that conductance increase is caused by synaptic input rather than voltage-activated conductances (Figure 8 in Berg and Ditlevsen, 2013). Application of strychnine had a strong depolarizing effect (Figure 1H) especially in the on-phase, which is also difficult to reconcile with the reciprocal E/I scheme (Berg et al., 2007; Vestergaard and Berg, 2015).

Contrary to the conclusions of GH2017, these observations suggest that a substantial fraction of the spinal neurons receive concurrent E/I, which may not exclude that others receive reciprocal. In fact, the neuronal population is divided between irregular and regular spiking, suggesting some receive reciprocal and others receive balanced input most likely on a spectrum between the two (Petersen and Berg, 2016; Berg, 2017). Notice in addition to the in-phase E/I there is also a weaker out-of-phase inhibition (Figure 1G). Spinal motor pattern generation may therefore be more complex and not exclusively conform to either of the schemes (Kishore et al., 2014).

## Author contributions

The author confirms being the sole contributor of this work and approved it for publication.

### Conflict of interest statement

The author declares that the research was conducted in the absence of any commercial or financial relationships that could be construed as a potential conflict of interest.

## References

[B1] Berg R. W. (2017). Neuronal population activity in spinal motor circuits: greater than the sum of its parts. Front. Neural Circ. 11:103. 10.3389/fncir.2017.00103 29311842 PMC5742103

[B2] Berg R. W. Alaburda A. Hounsgaard J. (2007). Balanced inhibition and excitation drive spike activity in spinal half-centers. Science 315, 390–393. 10.1126/science.1134960 17234950

[B3] Berg R. W. Ditlevsen S. (2013). Synaptic inhibition and excitation estimated via the time constant of membrane potential fluctuations. J. Neurophysiol. 110, 1021–1034. 10.1152/jn.00006.2013 23636725

[B4] Berg R. W. Ditlevsen S. Hounsgaard J. (2008). Intense synaptic activity enhances temporal resolution in spinal motoneurons. PLoS ONE 3:e3218. 10.1371/journal.pone.0003218 18795101 PMC2528963

[B5] Brette R. Destexhe A. (eds.). (2012). Intracellular recording, in Handbook of Neural Activity Measurement (New York, NY: Cambridge University Press), 44–91.

[B6] Chadderton P. Schaefer A. T. Williams S. R. Margrie T. W. (2014). Sensory-evoked synaptic integration in cerebellar and cerebral cortical neurons. Nat. Rev. Neurosci. 15, 71–83. 10.1038/nrn3648 24434910

[B7] Geertsen S. S. Stecina K. Meehan C. F. Nielsen J. B. Hultborn H. (2011). Reciprocal Ia inhibition contributes to motoneuronal hyperpolarisation during the inactive phase of locomotion and scratching in the cat. J. Physiol. 589, 119–134. 10.1113/jphysiol.2010.199125 21059756 PMC3039264

[B8] Guzulaitis R. Hounsgaard J. (2017). Synaptic excitation in spinal motoneurons alternates with synaptic inhibition and is balanced by outward rectification during rhythmic motor network activity. J. Neurosci. 37, 9239–9248. 10.1523/JNEUROSCI.0800-17.2017 28842417 PMC6596737

[B9] Johnston D. Wu S.-S. (1995). Foundations of Cellular Neurophysiology, Cambridge, MA: MIT Press.

[B10] Kishore S. Bagnall M. W. McLean D. L. (2014). Systematic shifts in the balance of excitation and inhibition coordinate the activity of axial motor pools at different speeds of locomotion. J. Neurosci. 34, 14046–14054. 10.1523/JNEUROSCI.0514-14.2014 25319701 PMC4198544

[B11] Kolind J. Hounsgaard J. Berg R. W. (2012). Opposing effects of intrinsic conductance and correlated synaptic input on Vm-fluctuations during network activity. Front. Comput. Neurosci. 6:40 10.3389/fncom.2012.00040 22783184 PMC3389371

[B12] Monier C. Fournier J. Frégnac Y. (2008). *In vitro* and *in vivo* measures of evoked excitatory and inhibitory conductance dynamics in sensory cortices. J. Neurosci. Methods 169, 323–365. 10.1016/j.jneumeth.2007.11.008 18215425

[B13] Petersen C. C. (2017). Whole-cell recording of neuronal membrane potential during behavior. Neuron 95, 1266–1281. 10.1016/j.neuron.2017.06.049 28910617

[B14] Petersen P. C. Berg R. W. (2016). Lognormal firing rate distribution reveals prominent fluctuation-driven regime in spinal motor networks. eLife 5:e18805. 10.7554/eLife.18805 27782883 PMC5135395

[B15] Petersen P. C. Vestergaard M. Jensen K. H. Berg R. W. (2014). Premotor spinal network with balanced excitation and inhibition during motor patterns has high resilience to structural division. J. Neurosci. 34, 2774–2784. 10.1523/JNEUROSCI.3349-13.2014 24553920 PMC6608521

[B16] Spruston N. Jaffe D. B. Williams S. H. Johnston D. (1993). Voltage- and space-clamp errors associated with the measurement of electrotonically remote synaptic events. J. Neurophysiol. 70, 781–802. 8410172 10.1152/jn.1993.70.2.781

[B17] Vestergaard M. Berg R. W. (2015). Divisive gain modulation of motoneurons by inhibition optimizes muscular control. J. Neurosci. 35, 3711–3723. 10.1523/JNEUROSCI.3899-14.2015 25716868 PMC6605555

[B18] Vich C. Berg R. W. Guillamon A. Ditlevsen S. (2017). Estimation of synaptic conductances in presence of nonlinear effects caused by subthreshold ionic currents. Front. Comput. Neurosci. 11:69. 10.3389/fncom.2017.00069 28790909 PMC5524927

[B19] Williams S. R. Mitchell S. J. (2008). Direct measurement of somatic voltage clamp errors in central neurons. Nat. Neurosci. 11, 790–798. 10.1038/nn.2137 18552844

